# Cohort-specific imputation of gene expression improves prediction of warfarin dose for African Americans

**DOI:** 10.1186/s13073-017-0495-0

**Published:** 2017-11-24

**Authors:** Assaf Gottlieb, Roxana Daneshjou, Marianne DeGorter, Stephane Bourgeois, Peter J. Svensson, Mia Wadelius, Panos Deloukas, Stephen B. Montgomery, Russ B. Altman

**Affiliations:** 1 School of Biomedical Informatics, University of Texas Health Center, 7000 Fannin St., Houston, TX 77030 USA; 2 Department of Genetics, Stanford University, Stanford, CA 94305 USA; 3 Department of Pathology, Stanford University, Stanford, CA 94305 USA; 4 William Harvey Research Institute, Barts and the London School of Medicine and Dentistry, Queen Mary University of London, London, EC1M 6BQ UK; 5 Department of Translational Medicine, University of Lund, Malmö, 205 02 Sweden; 6 Department of Medical Sciences and Science for Life laboratory, Uppsala University, Uppsala, 751 85 Sweden; 7 Princess Al-Jawhara Al-Brahim Centre of Excellence in Research of Hereditary Disorders (PACER-HD), King Abdulaziz University, Jeddah, 21589 Saudi Arabia; 8 Department of Bioengineering, Stanford University, Stanford, CA 94305 USA

**Keywords:** Pharmacogenomics, Warfarin dose, International Warfarin Pharmacogenetics Consortium, African Americans

## Abstract

**Background:**

Genome-wide association studies are useful for discovering genotype–phenotype associations but are limited because they require large cohorts to identify a signal, which can be population-specific. Mapping genetic variation to genes improves power and allows the effects of both protein-coding variation as well as variation in expression to be combined into “gene level” effects.

**Methods:**

Previous work has shown that warfarin dose can be predicted using information from genetic variation that affects protein-coding regions.

Here, we introduce a method that improves dose prediction by integrating tissue-specific gene expression. In particular, we use drug pathways and expression quantitative trait loci knowledge to impute gene expression—on the assumption that differential expression of key pathway genes may impact dose requirement. We focus on 116 genes from the pharmacokinetic and pharmacodynamic pathways of warfarin within training and validation sets comprising both European and African-descent individuals.

**Results:**

We build gene-tissue signatures associated with warfarin dose in a cohort-specific manner and identify a signature of 11 gene-tissue pairs that significantly augments the International Warfarin Pharmacogenetics Consortium dosage-prediction algorithm in both populations.

**Conclusions:**

Our results demonstrate that imputed expression can improve dose prediction and bridge population-specific compositions.

MATLAB code is available at https://github.com/assafgo/warfarin-cohort

**Electronic supplementary material:**

The online version of this article (doi:10.1186/s13073-017-0495-0) contains supplementary material, which is available to authorized users.

## Background

A crucial component to implementing precision medicine is elucidating how genetic variation affects drug response. These gene–drug associations can then be used for tailored drug selection and drug dosing [1, 2]. Genome-wide association studies (GWAS) allow the association of genetic variants like single nucleotide polymorphisms (SNPs) with a drug phenotype. While GWAS have successfully identified thousands of genotype–phenotype associations, they suffer from three limitations [3]: testing a large number of SNPs requires a large study cohort to identify a statistically significant signal; SNPs can be population-specific and findings from one population may not be applicable to another population [4]; and finally, finding the genes affected by phenotype-associated SNPs is challenging [5, 6].

Approaches that aggregate SNPs into genes or pathways have been developed to circumvent some of these drawbacks [7, 8]. Working within the gene or pathway level typically decreases the number of hypotheses [5] and may also bridge population-specific allelic compositions. Beyond direct measurement of genetic variation, approaches for using measured or imputed gene expression can potentially provide insight into biological mechanism [9]. For example, PrediXcan [10] imputes the expected baseline expression of a gene based on the allele composition of SNPs in proximity to that gene (*cis*-SNPs) and uses these predicted baseline expression values to identify gene associations to disease phenotypes.

**Fig. 1:**
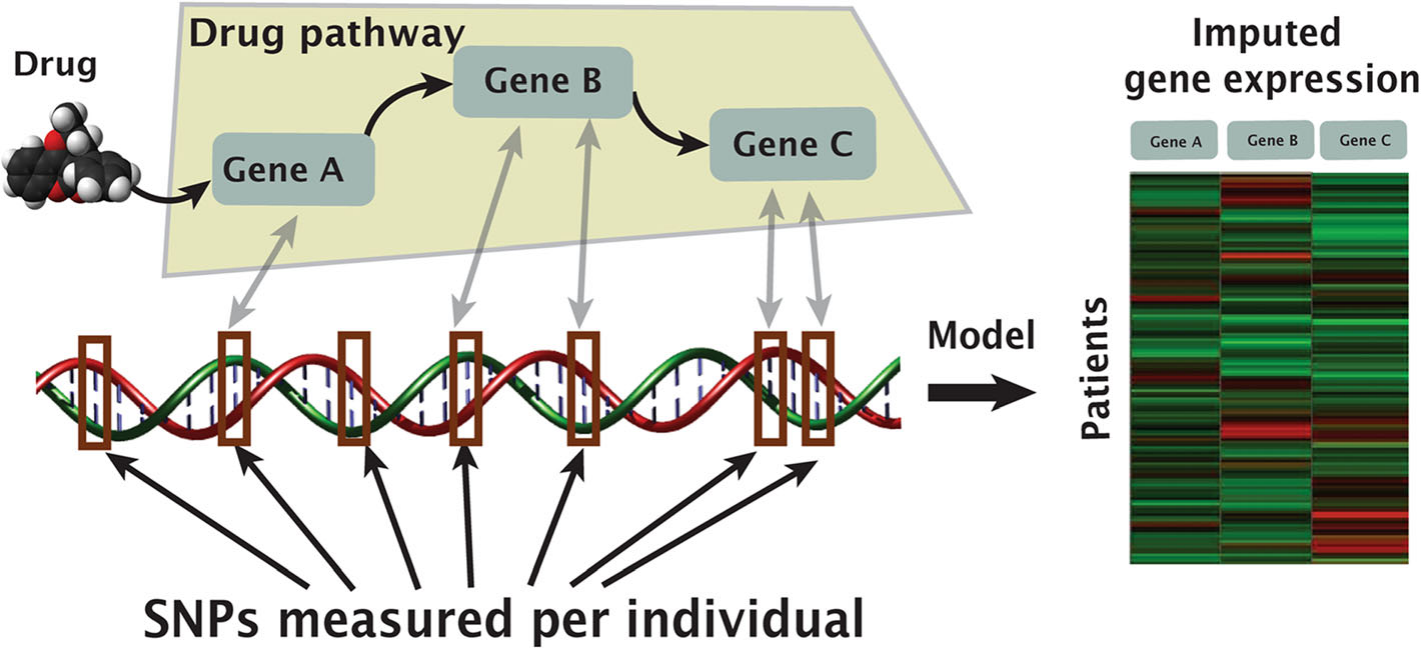
*Illustration* of the use of SNPs, measured in GWAS, to impute expression of drug-associated genes

One mechanism through which SNPs may affect drug response is by modulating the expression level of genes that are key for drug response. These SNP effects may be population- and/or tissue-specific, and the Genotype-Tissue Expression (GTEx) datasets [11] make it possible to assess tissue-specific baseline levels of gene expression for specific ancestries. In this work, we evaluate the degree to which estimation of baseline gene expression can improve prediction of drug response. We use the generic PrediXcan strategy in a modified way: (1) we impute gene expression in a manner that is cohort-specific; and (2) we impute genes only in tissues where expression quantitative loci (eQTLs) are associated with these genes. In order to have a more interpretable model, we focus on drug pathway genes relevant to the pharmacologic problem (see also [12]). We impute gene expression in specific tissues for each individual using the GTEx compendium [11] (Fig. 1) and learn a signature that is predictive of warfarin dose comprising gene-tissue pairs on a training cohort. We demonstrate the utility of the signatures by predicting warfarin dose in individuals of African American (AA) and European (EUR) descent. Warfarin dose prediction in African Americans is especially challenging, as the currently known genetic variations predict only a small amount of the dose variability [13, 14]. In both populations, our new signatures explain the additional 8–12% of the variance in warfarin dose that is unexplained by the International Warfarin Pharmacogenetics Consortium (IWPC) algorithm. We also associate 20 novel genes with warfarin dose, including two genes—LGALS2 and PLCG2—that are common to signatures of EUR and AA populations. Our method performs well on AA in contrast to the generic strategy. Through these improvements, we offer a general approach for prediction of drug response and discovery of associated gene candidates.

## Methods

Drug response is a complex phenotype and is regulated by multiple genes and across multiple tissues. To identify which genes (in the context of a tissue) might influence warfarin dosing, we: (1) imputed the expression of genes in warfarin pharmacokinetic (PK) and pharmacodynamic (PD) pathways using SNPs in proximity to each gene; (2) used the imputed gene-tissue pairs as features to learn a tissue-specific gene expression signature on the EUR or AA training cohort; and (3) validate the performance of the learned signature gene-tissue pairs on the EUR or AA validation cohorts relative to background models.

We compared a generic PrediXcan imputation of expression [10] with an approach that: (1) builds cohort-specific imputation models; and (2) imputes gene expression for each gene only in tissues where the gene expression has associated eQTLs. We learn the tissue-specific signatures on a EUR and AA cohorts and validate them by estimating warfarin dose on independent validation cohorts.

### Datasets

#### Gene-tissue expression data

Gene expression and eQTLs associated with 42 tissues were extracted from GTEx consortium version 6 [15] (excluding cell-lines of EBV-transformed lymphocytes and transformed fibroblasts; tissue statistics are available on the GTEx portal, http://www.gtexportal.org/home/tissueSummaryPage).

We imputed the expression of 116 genes assembled from the curated warfarin PD and PK pathways from PharmGKB [16] and from the predicted warfarin PD pathway genes [17] (Additional file 1: Table S1). Prediction of warfarin PD pathway was done by connecting curated warfarin-associated genes, including gene targets, pharmacogenes (genes associated with differential drug response) and disease genes, over the protein interaction network.

#### Training and validation cohorts

We selected a signature comprising gene-tissue pairs, predictive of warfarin dose, using a training cohort and validated the signature performance in predicting warfarin dose on a validation cohort. Table 1 lists cohort statistics and Additional file 1: Figure S1 displays cohort age distribution. The training set for the AA signature comprised 103 previously exome-sequenced individuals of AA descent which received either low dose (≤35 mg/week) or high dose (>49 mg/week) warfarin [18]. The EUR training cohort is the Cooper et al. dataset [19] with 180 genotyped individuals (five of which are of Hispanic origin), followed by imputation against HapMap. Validation was conducted on 225 genotyped individuals of AA descent, imputed against HapMap Phase 2 Release 22 [13, 20] and 233 genotyped individuals of EUR descent [21] imputed using HapMap3 (see Additional file 1: Figure S2 for distribution of doses in each study). The warfarin patient cohorts included 0.02–1.1% of missing values, which were imputed using *k*-nearest neighbors impute (*k* = 5). Based on 77 AA individuals, common to the training exome-sequenced cohort and the genotyped validation cohort, we were able to estimate 1% allelic mismatches (including missing values) between the two sequencing methods. These 77 individuals were subsequently excluded from the validation set.

**Table 1:** Cohort statistics

Study cohort	Patients (n)	Females (%)	Imputation method	Model SNPs (n)	Covered tissues (n)	Imputed genes (n)	Imputed gene-tissue pairs (n)
AA training	103	68	Generic	1309	39	112	901
			Cohort-specific	2175	41	96	539
AA validation	225	NA	Generic	5384	39	114	1727
			Cohort-specific	2480	41	96	539
EUR training	180	36	Generic	5305	39	114	1729
			Cohort-specific	2519	41	96	539
EUR validation	233	52	Generic	5313	39	114	1729
			Cohort-specific	2674	41	96	539

### Imputing tissue-specific gene expression

We imputed gene expression by building a regression model per gene in a given tissue from SNPs in cis with the gene, defined as closer than 1 Mb to the outer bounds of the gene using the GTEx compendium of genotypes and gene expression. We built a model for each gene-tissue pair using LASSO [22], either following the original PrediXcan methodology (the “generic” strategy) [10] or a “cohort-specific” strategy. For the generic PrediXcan imputation, we used the software package of PrediXcan with the weighted *cis*-SNPs in the PredictDB database [23] to generate imputed gene-tissue pairs.

**Fig. 2:**
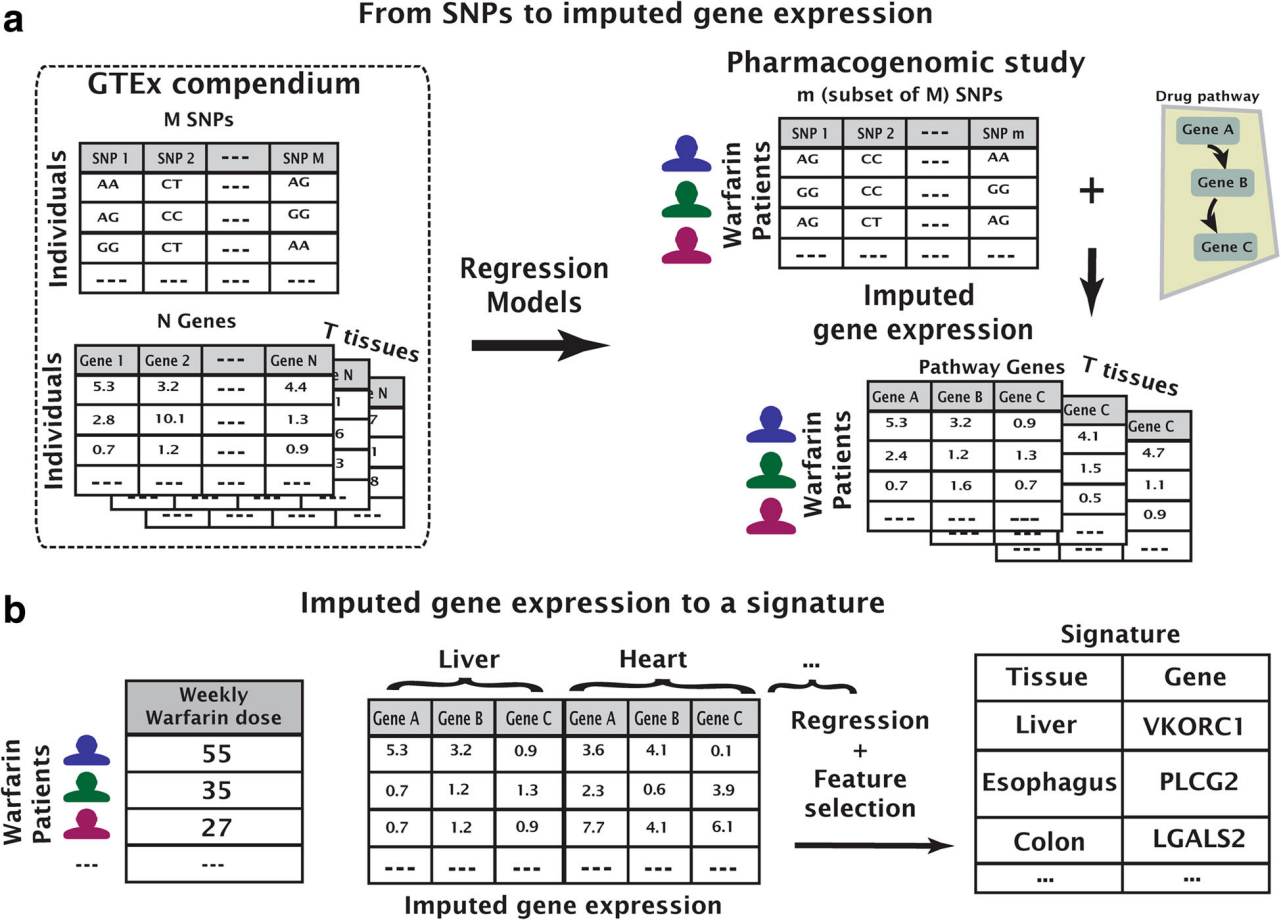
*Illustration* of the feature construction and signature selection methods. First, gene expression is imputed by regression models from cis-SNPs (**a**). Then, a signature is learned by regressing the drug response on the imputed expression features (**b**)

In the cohort-specific strategy, we followed the PrediXcan methodology to impute a gene using *cis*-SNPs (gene and SNP positions extracted from the human genome reference sequence GRCh38 and dbSNP build 144). Different from PrediXcan, we focused only on gene-tissue pairs where the gene has at least one significant eQTL (q-values ≤ 5%) in GTEx [11], resulting in 67,022 SNPs measured in any of the four warfarin studies and in cis with the warfarin-pathway genes. The four imputed warfarin GWAS contain only a partial set of the SNPs available in GTEx and partial overlap with each other. To bridge these differences in SNP content, our cohort-specific strategy was to build an independent model per cohort, based only on the SNPs measured in that cohort study (Fig. 2a). The gene expression regression models where computed with LASSO [22] using fivefold cross-validation to select the optimal regularization parameters.

Between 111 and 114 genes were imputed using the generic PrediXcan and 96 genes were imputed using the cohort-specific method (Table 1). Correspondingly, the number of model SNPs is smaller with the cohort-specific method except for the AA training cohort. The AA training cohort was measured with exome-sequencing array and shares smaller number of SNPs with the genotype arrays used in GTEx. Additional file 1: Figure S3 displays the relative overlap of *cis*-SNPS measured in each warfarin study that were used in imputing the genes.

Our method of validation was by comparing the performance of the generic and the cohort-specific strategies in predicting warfarin dose to background models (R^2^ measures), which we describe in the next sections. In order to gain insights into the differences between the imputation strategies, we also compared the imputed gene-tissue expression in two ways: (1) overlap of imputed genes across the four warfarin study cohorts for each imputation method independently (between-study similarity); and (2) the consistency of each study cohort across the two imputation strategies (between-strategy similarity). For the between-study similarity, we computed the mean imputed value of each gene-tissue pair across the study individuals and computed the Pearson correlation between the gene-tissue pairs’ means across the cohorts. For the between-strategy similarity, we computed the correlations of each gene-tissue pair across individual patients and averaged across all gene-tissue pair. All computations were performed using MATLAB v8.4. MATLAB code is available at https://github.com/assafgo/warfarin-cohort and the imputed data in Additional file 2.

### Learning signatures for warfarin dose

Imputed gene-tissue expression values were computed per warfarin patient, using all GTEx data for the 39–41 tissues, resulting in 539–1727 gene-tissue pairs serving as features (Table 1). We assume that variation in gene expression affects warfarin dose, but this effect might involve only a subset of the warfarin pathway genes and could be tissue-specific. We thus learned a signature, comprising gene-tissue pairs, that is predictive of warfarin dose using the EUR and AA training cohorts. Gene-tissue signatures were learned using LASSO regression analysis with fivefold cross-validation, selecting the shrinkage parameter which provided the minimal mean square error (Fig. 2b). For robustness, we repeated this procedure 100 times with different random cross-validation partitions and selected for the signature gene-tissue pairs appearing in more than half of the repeats.

### Predicting warfarin dose

We measured the performance of the signatures based on the R^2^ of the unexplained portion of the IWPC algorithm. Specifically, we computed the predicted dose according to the IWPC dosing algorithm for each validation cohort and inferred the residual dose, i.e. the difference between an individual’s actual therapeutic dose and the IWPC predicted dose. In the AA validation dataset, the IWPC dose was computed without the information regarding use of enzyme reducers (phenytoin, carbamazepine, or rifampin), which could potentially change the predicted dose by up to 9% [20].

Signature performance was evaluated relative to two background models: (1) a shuffled model, in which the residual dose was shuffled 10,000 times; and (2) a random signatures model, created from 10,000 equal-sized randomly selected signatures, chosen from the gene-tissue pairs not in the signature. *P* values of regression R^2^ values were empirically computed relative to these background models and corrected for false discovery rate (FDR) [24] of 0.05 across the tested signatures.

## Results

We tested whether a modified PrediXcan approach could improve inference of warfarin dose for EUR and AA populations by imputing GWAS and constructing population-specific signatures. We compared the generic PrediXcan approach and the modified, cohort-specific, version based on their ability to improve the gold standard IWPC dosing algorithm.

### Imputing tissue-specific gene expression

The first step involved creating features from imputed gene tissue. We assessed the differences between the two imputation strategies through between-study and between-strategy tests (see “Methods”).

The between-study similarity shows the imputed gene expression distributions across studies. It was high between the EUR training and validation cohorts for both the generic and cohort-specific imputation strategies (Pearson ρ = 0.96, *p* ~ 0 and ρ = 0.72, *p* < e^–85^, respectively). It was significant between the AA training and validation cohorts only for the cohort-specific method (Pearson ρ = 0.54, *p* < 2e^–41^, Additional file 1: Figure S4).

The between-strategy similarity displays moderate similarities between the two imputation methods–average Pearson correlations between imputed genes are in the range of 0.52–0.59 (±0.39) across the AA and EUR cohorts, with slightly higher similarities across the EUR cohorts.

### Learning gene-tissue signatures for warfarin dose

We next learned separate signatures using the generic and the cohort-specific imputation strategies. The generic strategy produced a EUR signature with 16 gene-tissue pairs but failed to produce a signature on the AA training cohort. In contrast, the cohort-specific signatures produced a EUR signature comprising 11 gene-tissue pairs and an AA signature comprising 17 gene-tissue pairs (Table 2).

**Table 2:** Predictive signatures for warfarin dose

Cohort-specific				Generic^a^	
African American		Central European		Central European	
Gene	Tissue	Gene	Tissue	Gene	Tissue
CYP1A1	Adipose, subcutaneous	ARRB1	Adipose, subcutaneous	CCND1	Adrenal gland
AKT1	Pancreas	CUBN	Adrenal gland	ABL1	Skin not-sun-exposed suprapubic
ALOX5	Adrenal gland; brain, cortex	GCLC	Spleen	ATF2	Brain, cerebellar hemisphere; brain, cerebellum
EPHX1	Heart, left ventricle; thyroid	GGCX	Brain, cerebellar hemisphere	AURKA	Brain, frontal cortex BA9
GNAI2	Whole blood	LGALS2	Pancreas	CCND1	Esophagus mucosa
ITGB1	Tibial nerve	PLCG2	Esophagus, muscularis	CUBN	Adrenal gland
LGALS2	Colon transverse	PSMA6	Skeletal muscle	CYP2C18	Liver
NCOA1	Brain, caudate basal ganglia	PTK2	Skeletal muscle	GCLM	Brain, cerebellum
PLCG2	Esophagus, muscularis; pancreas	UBE2I	Esophagus, muscularis	ITGA2B	Thyroid
PROZ	Spleen	VKORC1	Liver; thyroid	JUN	Tibial artery
SERPINF2	Thyroid			MGP	Esophagus gastroesophageal junction
SMAD3	Esophagus, mucosa			PSEN1	Pituitary
STX4	Colon transverse			SMAD2	Testis
VKORC1	Liver			VKORC1	Thyroid; heart atrial appendage

^a^The generic methods did not produce a signature on the AA training cohort

### Predicting warfarin dose

We estimated the signatures performance on the validation cohorts by regressing the signature gene-tissue pairs against the residuals of the IWPC algorithm. We report here the R^2^ explaining the variance of these residuals.

#### Signatures for patients of EUR descent

**Fig. 3:**
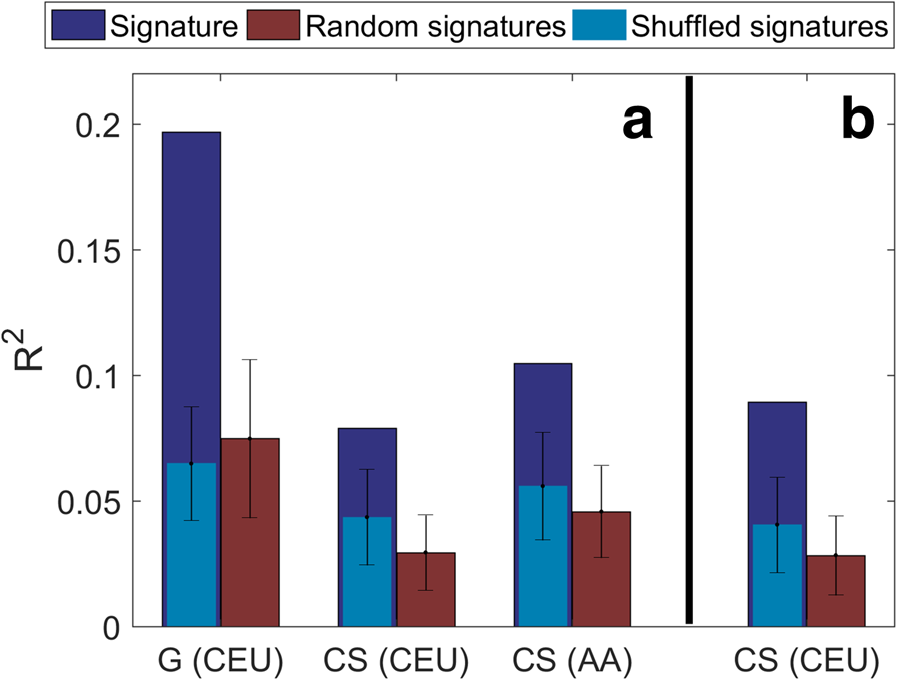
R^2^ results of the predicted unexplained variance in warfarin dose by the IWPC algorithm for the EUR (**a**) and AA (**b**) validation cohorts. Represented are the signatures (*dark blue*), random signatures (*red*), and signatures on shuffled data (*light blue*) as the background models for the AA and EUR signatures. EUR and AA in *parentheses* are the training cohort for the signature; *G* generic imputation method, *CS* cohort-specific imputation

Both the generic and the cohort-specific strategies produced signatures that performed better than the background, with the generic strategy obtaining higher R^2^ results on the EUR validation set (R^2^ = 0.2, *p* < 0.008 and R^2^ = 0.08, *p* < 0.05 for generic and cohort-specific strategies, respectively; Table 3, Fig. 3a). The cohort-specific EUR signature performed well also on the AA validation set (R^2^ = 0.09, *p* < 0.02; Table 3, Fig. 3b) while the generic EUR signature was not better than the background.

**Table 3:** Performance of the generic and cohort-specific signatures on different warfarin studies

Validation cohort	Signature	R^2^ regression against IWPC residuals	*P* value of difference from the background^a^
EUR validation	EUR, generic	0.2	**0.008** (**e** ^**–4**^)
	EUR, cohort-specific	0.08	**0.004** (0.05)
	AA, cohort-specific	0.1	**0.004** (**0.03**)
AA validation	EUR, cohort-specific	0.09	**0.0009** (**0.02**)

The R^2^ coefficient is measured on the IWPC residuals

*P* values below FDR of 0.05 are bolded

^a^Background computed as random signatures. *P* values of shuffled signatures in parentheses

#### Signatures for patients of AA descent

The generic imputation strategy failed to produce a signature on the AA training cohort. While the cohort-specific AA-trained signature was not better than background on the AA validation set, it showed significant improvement on both the EUR validation and EUR training cohorts (R^2^ = 0.1, *p* < 0.01 and R^2^ = 0.24, *p* < 0.0001 for the EUR validation and training, respectively; Table 3, Fig. 3a).

### Analysis of gene-tissue pair signatures

The imputed expression of three tissue-gene pairs displayed significant correlation with warfarin dose in the EUR validation cohort: (1) VKORC1 in liver and in thyroid (Pearson correlation, ρ = 0.49, *p* < e^–15^ in liver and ρ = 0.28, *p* < e^–5^ in thyroid); (2) STX4 in transverse colon (ρ = 0.3, *p* < 2e^–6^); and (3) CYP2C18 in liver (ρ = 0.35, *p* < 3e^–8^). Out of these three, VKORC1 in liver is the only single predictor of dose that is better than the background (R^2^ = 0.03, *p* < 0.005 for the cohort-specific imputed expression and R^2^ = 0.03, *p* < 0.02 for the generic). No AA signature gene-tissue pairs displayed individually significant correlation to warfarin dose.

Three genes are common to the EUR and AA cohort-specific signatures (Table 2): VKORC1 in liver; PLCG2 in muscularis esophagus; and LGALS2 in pancreas (EUR signature) and transverse colon in (AA signature). CUBN in adrenal gland is common to the EUR generic and EUR cohort-specific signatures.

## Discussion

We have introduced a strategy to use imputed gene expression in the context of drug pathways to select a signature of gene-tissue pairs predictive of warfarin dose. By focusing on drug response-associated genes, we increase the likelihood of finding biologically relevant variants that influence transcriptional regulation in the context of a drug and reduce the size of the hypothesis space. We used the LASSO linear model to provide an interpretable model signature, noting that the LASSO with non-linear measurements is equivalent to one with linear measurements [25]. We compared the performance of the current state of the art, PrediXcan, to a modified, cohort-specific strategy on patients of AA and EUR descent.

We evaluated our performance by inferring the residual variation of the IWPC algorithm. The generic strategy better explained warfarin dose than our modified method on individuals of EUR descent, but performed poorly on African Americans. This is not surprising, given the preponderance of EUR-descent individuals used to generate the imputation model. Indeed, the population sampled in GTEx includes 84% white and only 14% African Americans. The results are consistent with previous findings that warfarin dose models trained on individuals of EUR descent have poor performance on African Americans [13, 26–28]. Since the AA training cohort was measured using exome-sequencing while the other studies used genotyping, it is possible that these platform differences also affected the ability of the generic strategy to perform well on the AA cohort as the performance of the cohort-specific AA signature to perform better than the EUR signature on the AA validation set.

While the cohort-specific strategy performed worse on the EUR population, its signatures generalized better across populations and sequencing platforms, where EUR-trained signatures performing well on African Americans and vice versa, explaining an R^2^ of 0.08–0.1 of the variability over the IWPC algorithm. Our results suggest that cohort-specific models are advantageous in cases where the set of SNPs measured for the cohort differ from the set used for building the models and calls for diversifying the sampled populations in GTEx to produce better pharmacogenomic models. As the gender and age distributions in GTEx (34% women and majority of individuals aged 50–70 years) differ from the distributions in the warfarin cohorts (Table 1 and Additional file 1: Figure S1), we estimate that models that further stratify the GTEx population based on these covariates may produce more accurate imputation models and improve the pharmacogenomics models.

Warfarin acts as an inhibitor of VKORC1 and is part of the IWPC dose algorithm. Polymorphisms in its cis-SNP rs9923231 account for approximately 25% of the variance in stabilized warfarin dose and is currently considered the single largest predictor of warfarin dose [29]. While the generic and cohort-specific models for VKORC1, in both the AA and EUR training cohorts, do not include this SNP, they include SNPs in linkage disequilibrium with it, such as rs2855475, rs2359612, and rs749767 (R^2^ > 0.96) along with 13–31 additional SNPs in both models. It is encouraging that imputed expression of VKORC1 in liver was found to be a strong dose predictor, suggesting that a significant component in VKORC1 effect on dose is through transcriptional regulation.

Only three genes, VKORC1, STX4, and CYP2C18, are individually correlated with warfarin dose, accounting together for less than one-third of the entire signature explained dose (R^2^ = 0.035, *p* < 0.02 on the EUR cohort and insignificant on the AA cohort), which suggests that gene expression associated with dose is combinatorial in nature and supports our pathway-directed multivariate analysis methodology. Specifically, STX4 resides close to VKORC1 (~50 Kb apart) and some of the warfarin-associated SNPs like rs10871454 [19] could be attributed to either genes or affect both gene expression. Additionally, the cohort-specific signatures include genes with known polymorphism associated with warfarin dose such as GGCX, within African Americans [30] and EPHX1 within Caucasians [31]. Lastly, CYP2C18, appearing in the generic signature, was previously reported to be associated with warfarin dose [32]. The report explained this association by linkage disequilibrium of CYP2C18-associated SNP rs7896133 with CYP2C9*3 (PredictDB does not include a CYP2C9 model in liver). rs7896133 is indeed moderately linked to CYP2C9*3 (r^2^ = 0.68, using SNAP tool [33]) and is included in PredictDB models for CYP2C18 in liver but the generic models include additional eight cis-SNPs, four of them (rs7067881, rs9332214, rs7920801, and rs7088784) with similar or larger weights than rs7896133 and only two of these in LD with CYP2C9*3 (rs9332214 and rs7088784, r^2^ = 0.87 and 0.68, respectively), suggesting that CYP2C18 might have another mechanism of association with warfarin dose. Notably, rs12777823, associated with warfarin dose in African Americans and independent of CYP2C9*2 and CYP2C9*3 [34] was not measured in any of the four cohorts in our study and was also not part of the generic models in PredictDB.

Liver is associated with two (out of three) gene-tissue pairs correlated with warfarin dose (VKORC1 and CYP2C18). Liver indeed plays a role in the metabolism of warfarin [35]. We have not a priori filtered tissues based on estimated relevance to warfarin response. Our signatures thus include tissues that are not typically considered relevant to warfarin’s mode of action. The reasons may be that these expression values in the selected tissues may correlate with expression in other relevant tissues or may represent genuine evidence of an unexpected role of new tissues in warfarin response. Tissue-specific knowledge may improve our methodology and should be considered in follow-up work, taking into account also tissue-specific detection sensitivity [15].

## Conclusions

Imputation of gene expression for genes relevant to drug action can increase the power of GWAS in explaining drug response such as warfarin dose. Focusing on genes with genetically driven (vs environmentally driven) expression in the form of eQTLs allows us to build models of drug response that reflect the expected effects of critical genes in particular tissues. We have further shown that these models can be computed using a cohort-specific strategy to improve their predictive power.

We chose to use previous knowledge in the form of pre-selected genes in order to avoid overfitting and for interpretability of the results. However, imputing all the genes can potentially improve the model further and identify novel genes associated with warfarin dose. For the same reason of interpretability, we used the LASSO linear regression model that results in relatively short and robust signatures, notably showing that the LASSO with non-linear measurements is in many cases equivalent to one with linear measurements [25]. Nevertheless, it would be worthwhile in future works to compare the performance of other non-linear regression models in predicting warfarin dose.

## Additional files

Additional file 1: Supplementary figures and tables. (PDF 724 kb)

Additional file 2: A zip files containing the four imputed data files WarfarinData.zip. (ZIP 997 kb)

## Abbreviations

AA: African American
eQTL: Expression quantitative loci
EUR: European
FDR: False discovery rate
GTEx: Genotype-tissue expression
GWAS: Genome-wide association studies
IWPC: International warfarin pharmacogenetics consortium
PD: Pharmacodynamic
PK: Pharmacokinetic
SNP: Single nucleotide polymorphisms
