# Supplementary material for: Cohort-specific imputation of gene expression improves prediction of warfarin dose for African Americans
Source: Genome Med. 2017 Nov 24;9:98. doi: 10.1186/s13073-017-0495-0 (PMC5702158; doi:10.1186/s13073-017-0495-0)
Supplement: Supplementary file 1 — Supplementary figures and tables. (PDF 724 kb) [file 13073_2017_495_MOESM1_ESM.pdf]

Cohort-specific imputation of gene expression improves prediction of warfarin dose for African Americans

Supplementary information

Assaf Gottlieb, PhD [1*], Roxana Daneshjou, MD, PhD [2], Marianne DeGorter, PhD [2,3], , Stephane Bourgeois, MsC [4], Peter J. Svensson, MD, PhD [5], Mia Wadelius, MD, PhD [6], Panos Deloukas , PhD [4,7], Stephen B. Montgomery, PhD [2,3] & Russ B. Altman, MD, PhD [2,8,]

[1] *School of Biomedical Informatics, University of Texas Health Center, Houston, 77030*

[2] *Department of Genetics, Stanford University, Stanford, 94305*

[3] *Department of Pathology, Stanford University, Stanford, 94305*

[4] *William Harvey Research Institute, Barts and the London School of Medicine and Dentistry, Queen Mary University of London, London, United Kingdom, EC1M 6BQ*

[5] *Department of Translational Medicine, University of Lund, Malmö, Sweden, SE-205 02*

[6] *Department of Medical Sciences and Science for Life laboratory, Uppsala University, Uppsala, Sweden, SE-751 85*

[7] *Princess Al-Jawhara Al-Brahim Centre of Excellence in Research of Hereditary Disorders (PACER-HD), King Abdulaziz University, Jeddah, Saudi Arabia, 21589*

[8] *Department of Bioengineering, Stanford University, Stanford, 94305*

Supplementary Figures

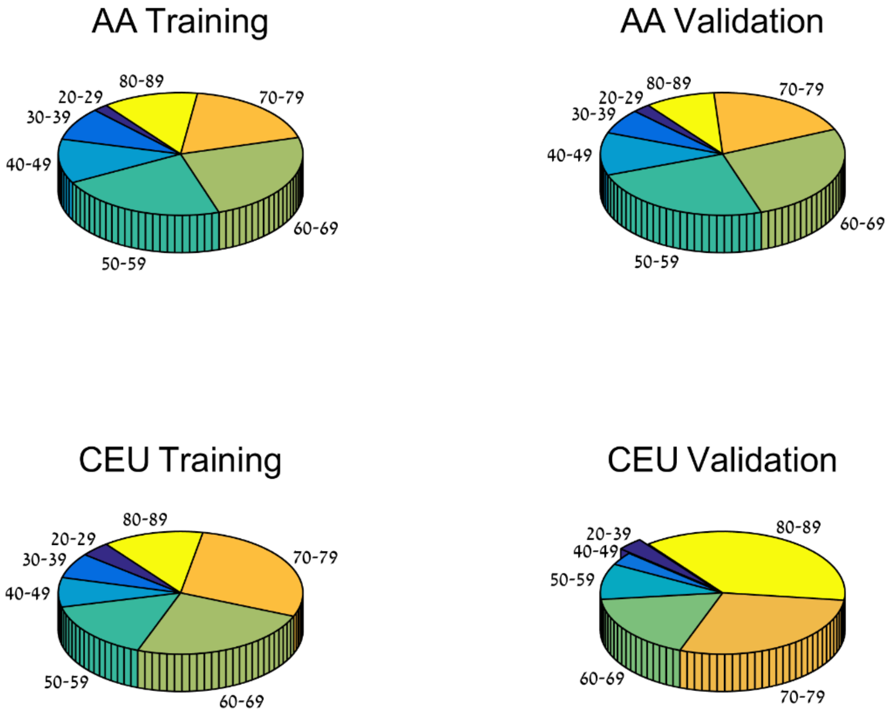

Figure S1: Age distribution for each of the warfarin study cohorts.

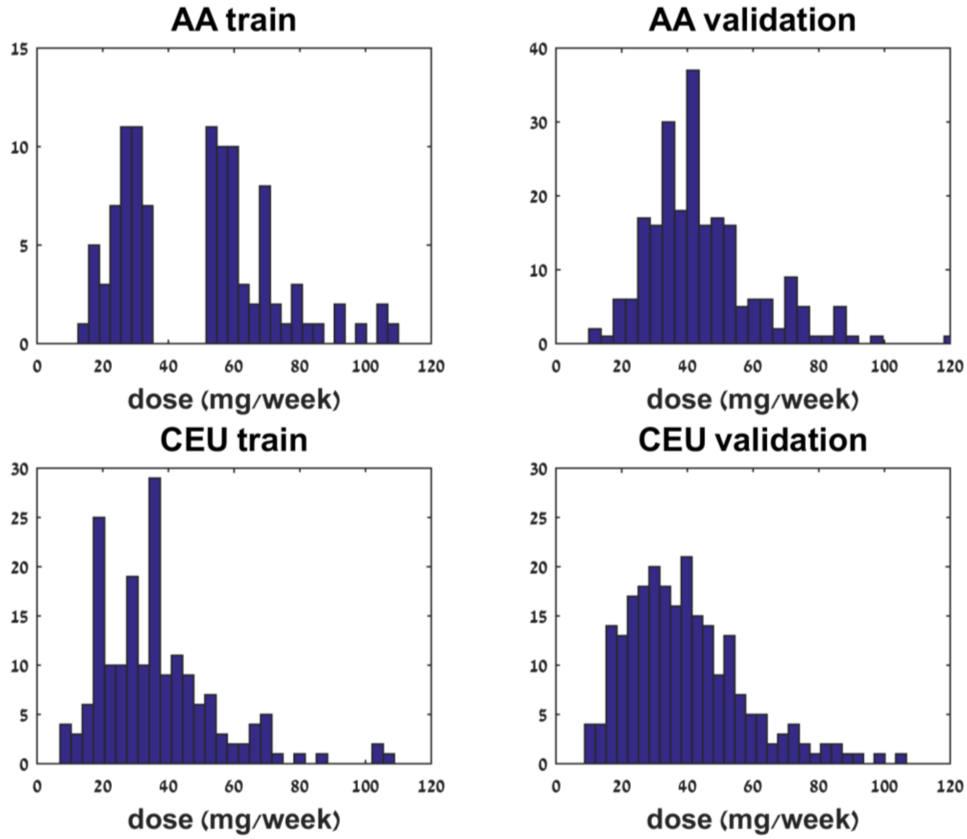

Figure S2: Weekly dose distribution or each of the warfarin study cohorts.

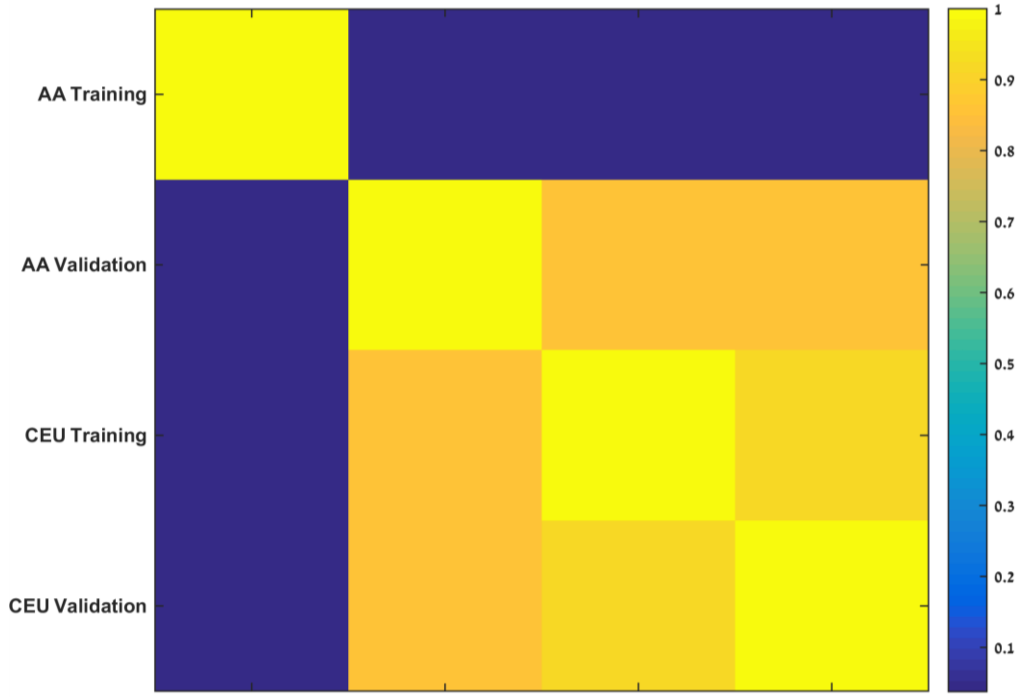

Figure S3: Relative overlap of measured cis-SNPs between cohorts (Jaccard score).

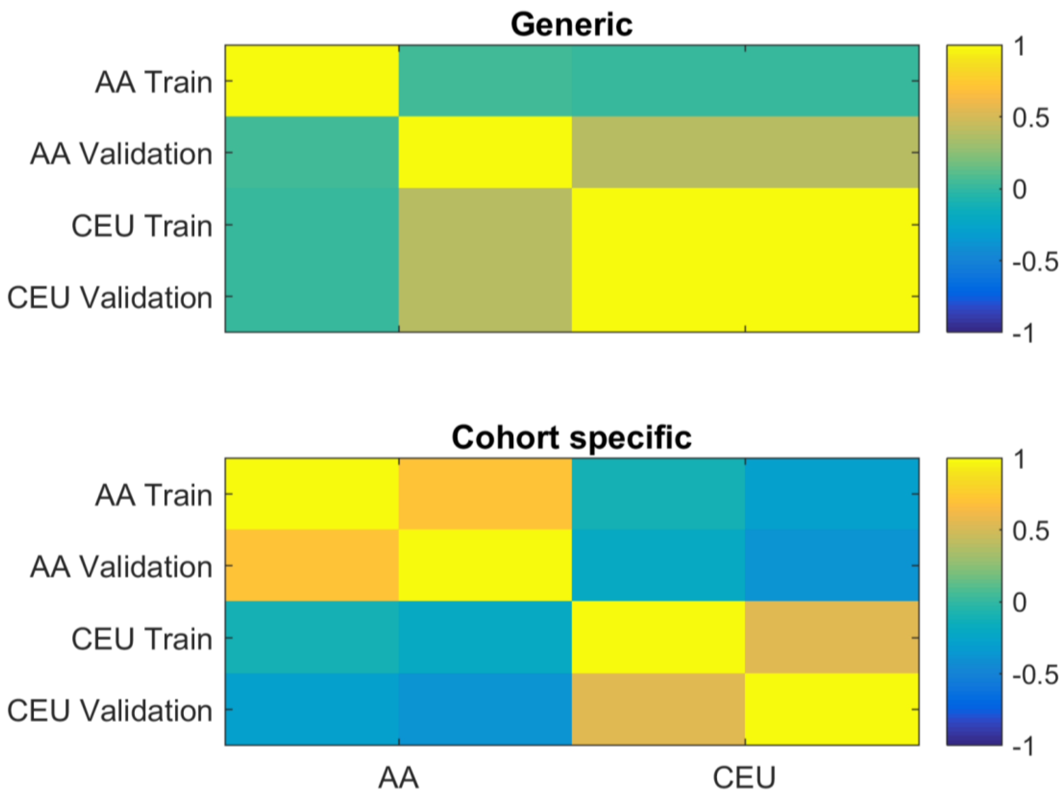

Figure S4: Pearson correlations between imputed gene expression means across cohorts for the generic and cohort-specific imputation methods.

Supplementary Tables

Table S1. **List of warfarin-associated genes**

Warfarin relation	Gene Name	Ensemble ID	Gene Description
Curated	ABCB1	ENSG00000085563	ATP-binding cassette, sub-family B (MDR/TAP), member 1
Inferred	ABL1	ENSG00000097007	c-abl oncogene 1, receptor tyrosine kinase
Inferred	ADCY9	ENSG00000162104	adenylate cyclase 9
Inferred	AKT1	ENSG00000142208	v-akt murine thymoma viral oncogene homolog 1
Inferred	ALOX5	ENSG00000012779	arachidonate 5-lipoxygenase
Curated	ALOX5AP	ENSG00000132965	arachidonate 5-lipoxygenase-activating protein
Inferred	ARRB1	ENSG00000137486	arrestin, beta 1
Inferred	ARRB2	ENSG00000141480	arrestin, beta 2
Inferred	ATF2	ENSG00000115966	activating transcription factor 2
Inferred	AURKA	ENSG00000087586	aurora kinase A; aurora kinase A pseudogene 1
Inferred	BCL2	ENSG00000171791	B-cell CLL/lymphoma 2
Inferred	BDKRB2	ENSG00000168398	bradykinin receptor B2
Curated	BGLAP	ENSG00000160783	bone gamma-carboxyglutamate (gla) protein; polyamine-modulated factor 1
Inferred	C1QBP	ENSG00000108561	complement component 1, q subcomponent binding protein
Curated	CALU	ENSG00000128595	calumenin
Inferred	CAV1	ENSG00000105974	caveolin 1, caveolae protein, 22kDa
Inferred	CCND1	ENSG00000110092	cyclin D1
Inferred	CDK2	ENSG00000123374	cyclin-dependent kinase 2
Inferred	CDK4	ENSG00000135446	cyclin-dependent kinase 4
Inferred	CDK5	ENSG00000164885	cyclin-dependent kinase 5
Inferred	CHUK	ENSG00000213341	conserved helix-loop-helix ubiquitous kinase
Inferred	CRKL	ENSG00000099942	v-crk sarcoma virus CT10 oncogene homolog (avian)-like
Inferred	CSNK2A1	ENSG00000101266	casein kinase 2, alpha 1 polypeptide pseudogene; casein kinase 2, alpha 1 polypeptide
Inferred	CSNK2B	ENSG00000204435	lymphocyte antigen 6 complex, locus G5B; casein kinase 2, beta polypeptide
Inferred	CTNNB1	ENSG00000168036	catenin (cadherin-associated protein), beta 1, 88kDa
Inferred	CUBN	ENSG00000107611	cubilin (intrinsic factor-cobalamin receptor)
Curated	CYP1A1	ENSG00000140465	cytochrome P450, family 1, subfamily A, polypeptide 1
Inferred	CYP2B6	ENSG00000197408	cytochrome P450, family 2, subfamily B, polypeptide 6
Curated	CYP2C18	ENSG00000108242	cytochrome P450, family 2, subfamily C, polypeptide 18

Curated	CYP2C19	ENSG00000165841	cytochrome P450, family 2, subfamily C, polypeptide 19
Curated	CYP2C8	ENSG00000138115	cytochrome P450, family 2, subfamily C, polypeptide 8
Curated	CYP2C9	ENSG00000138109	cytochrome P450, family 2, subfamily C, polypeptide 9
Curated	CYP3A4	ENSG00000160868	cytochrome P450, family 3, subfamily A, polypeptide 4
Inferred	CYP3A43	ENSG00000021461	cytochrome P450, family 3, subfamily A, polypeptide 43
Inferred	DAB1	ENSG00000173406	disabled homolog 1 (Drosophila)
Inferred	DLL1	ENSG00000198719	delta-like 1 (Drosophila)
Inferred	DVL1	ENSG00000107404	dishevelled, dsh homolog 1 (Drosophila); dishevelled, dsh homolog 1 (Drosophila)-like 1
Inferred	DVL2	ENSG00000004975	dishevelled, dsh homolog 2 (Drosophila)
Inferred	EGFR	ENSG00000146648	epidermal growth factor receptor (erythroblastic leukemia viral (v-erb-b) oncogene homolog, avian)
Inferred	EP300	ENSG00000100393	E1A binding protein p300
Curated	EPHX1	ENSG00000143819	epoxide hydrolase 1, microsomal (xenobiotic)
Curated	ESR1	ENSG00000091831	estrogen receptor 1
Inferred	ETS1	ENSG00000134954	v-ets erythroblastosis virus E26 oncogene homolog 1 (avian)
Curated	F10	ENSG00000126218	coagulation factor X
Curated	F13A1	ENSG00000124491	coagulation factor XIII, A1 polypeptide
Inferred	F2R	ENSG00000181104	coagulation factor II (thrombin) receptor
Inferred	F2RL2	ENSG00000164220	coagulation factor II (thrombin) receptor-like 2
Curated	F5	ENSG00000198734	coagulation factor V (proaccelerin, labile factor)
Curated	F7	ENSG00000057593	coagulation factor VII (serum prothrombin conversion accelerator)
Inferred	FGA	ENSG00000171560	fibrinogen alpha chain
Inferred	FN1	ENSG00000115414	fibronectin 1
Curated	GAS6	ENSG00000183087	similar to growth arrest-specific 6; growth arrest-specific 6
Curated	GCLC	ENSG00000001084	glutamate-cysteine ligase, catalytic subunit
Curated	GCLM	ENSG00000023909	glutamate-cysteine ligase, modifier subunit
Curated	GGCX	ENSG00000115486	gamma-glutamyl carboxylase
Inferred	GNA13	ENSG00000120063	guanine nucleotide binding protein (G protein), alpha 13
Inferred	GNAI2	ENSG00000114353	guanine nucleotide binding protein (G protein), alpha inhibiting activity polypeptide 2
Inferred	GRB2	ENSG00000177885	growth factor receptor-bound protein 2
Inferred	GSS	ENSG00000100983	glutathione synthetase
Inferred	HNF1A	ENSG00000135100	HNF1 homeobox A
Inferred	HNF4A	ENSG00000101076	hepatocyte nuclear factor 4, alpha

Inferred	HSPA4	ENSG00000170606	heat shock 70kDa protein 4
Curated	HTRA1	ENSG00000166033	HtrA serine peptidase 1
Inferred	ITGA2B	ENSG00000005961	integrin, alpha 2b (platelet glycoprotein IIb of IIb/IIIa complex, antigen CD41)
Inferred	ITGA4	ENSG00000115232	integrin, alpha 4 (antigen CD49D, alpha 4 subunit of VLA-4 receptor)
Inferred	ITGB1	ENSG00000150093	integrin, beta 1 (fibronectin receptor, beta polypeptide, antigen CD29 includes MDF2, MSK12)
Curated	ITGB3	ENSG00000056345	integrin, beta 3 (platelet glycoprotein IIIa, antigen CD61)
Inferred	JUN	ENSG00000177606	jun oncogene
Curated	KCNE2	ENSG00000159197	potassium voltage-gated channel, Isk-related family, member 2
Curated	KCNQ1	ENSG00000053918	potassium voltage-gated channel, KQT-like subfamily, member 1
Inferred	KNG1	ENSG00000113889	kininogen 1
Curated	LGALS2	ENSG00000100079	lectin, galactoside-binding, soluble, 2
Curated	LRP8	ENSG00000157193	low density lipoprotein receptor-related protein 8, apolipoprotein e receptor
Inferred	LRPAP1	ENSG00000163956	low density lipoprotein receptor-related protein associated protein 1
Inferred	MAPK1	ENSG00000100030	mitogen-activated protein kinase 1
Curated	MGP	ENSG00000111341	matrix Gla protein
Inferred	MMP9	ENSG00000100985	matrix metallopeptidase 9 (gelatinase B, 92kDa gelatinase, 92kDa type IV collagenase)
Inferred	MYC	ENSG00000136997	v-myc myelocytomatosis viral oncogene homolog (avian)
Inferred	NCOA1	ENSG00000084676	nuclear receptor coactivator 1
Curated	NOS3	ENSG00000164867	nitric oxide synthase 3 (endothelial cell)
Curated	NOTCH3	ENSG00000074181	Notch homolog 3 (Drosophila)
Inferred	PAFAH1B1	ENSG00000007168	platelet-activating factor acetylhydrolase, isoform Ib, subunit 1 (45kDa)
Curated	PDE4D	ENSG00000113448	phosphodiesterase 4D, cAMP-specific (phosphodiesterase E3 dunce homolog, Drosophila)
Inferred	PLA2G4A	ENSG00000116711	phospholipase A2, group IVA (cytosolic, calcium-dependent)
Inferred	PLCG2	ENSG00000197943	phospholipase C, gamma 2 (phosphatidylinositol-specific)
Inferred	PRKACA	ENSG00000072062	protein kinase, cAMP-dependent, catalytic, alpha
Inferred	PRKCA	ENSG00000154229	protein kinase C, alpha
Inferred	PRKCB	ENSG00000166501	protein kinase C, beta
Curated	PRKCH	ENSG00000027075	protein kinase C, eta
Curated	PROC	ENSG00000115718	protein C (inactivator of coagulation factors Va and VIIIa)
Curated	PROS1	ENSG00000184500	protein S (alpha)

Curated	PROZ	ENSG00000126231	protein Z, vitamin K-dependent plasma glycoprotein
Inferred	PSEN1	ENSG00000080815	presenilin 1
Inferred	PSMA5	ENSG00000143106	proteasome (prosome, macropain) subunit, alpha type, 5
Curated	PSMA6	ENSG00000100902	proteasome (prosome, macropain) subunit, alpha type, 6
Inferred	PTGS2	ENSG00000073756	prostaglandin-endoperoxide synthase 2 (prostaglandin G/H synthase and cyclooxygenase)
Inferred	PTK2	ENSG00000169398	PTK2 protein tyrosine kinase 2
Inferred	RAC1	ENSG00000136238	ras-related C3 botulinum toxin substrate 1 (rho family, small GTP binding protein Rac1)
Inferred	RAF1	ENSG00000132155	v-raf-1 murine leukemia viral oncogene homolog 1
Inferred	SERPINF2	ENSG00000167711	serpin peptidase inhibitor, clade F (alpha-2 antiplasmin, pigment epithelium derived factor), member 2
Inferred	SMAD2	ENSG00000175387	SMAD family member 2
Inferred	SMAD3	ENSG00000166949	SMAD family member 3
Inferred	SMAD4	ENSG00000141646	SMAD family member 4
Inferred	SNAP25	ENSG00000132639	synaptosomal-associated protein, 25kDa
Inferred	SRC	ENSG00000197122	v-src sarcoma (Schmidt-Ruppin A-2) viral oncogene homolog (avian)
Curated	STX4	ENSG00000103496	syntaxin 4
Inferred	TFPI	ENSG00000003436	tissue factor pathway inhibitor (lipoprotein-associated coagulation inhibitor)
Inferred	THBS1	ENSG00000137801	thrombospondin 1
Inferred	TNFRSF1A	ENSG00000067182	tumor necrosis factor receptor superfamily, member 1A
Inferred	TNFRSF1B	ENSG00000028137	tumor necrosis factor receptor superfamily, member 1B
Curated	TNFSF4	ENSG00000117586	tumor necrosis factor (ligand) superfamily, member 4
Inferred	TP53	ENSG00000141510	tumor protein p53
Inferred	TRAF6	ENSG00000175104	TNF receptor-associated factor 6
Inferred	TUBA1A	ENSG00000167552	tubulin, alpha 1a
Inferred	UBE2I	ENSG00000103275	ubiquitin-conjugating enzyme E2I (UBC9 homolog, yeast)
Curated	VKORC1	ENSG00000167397	vitamin K epoxide reductase complex, subunit 1
